# Body-Specific Attention to the Hands and Feet in Healthy Adults

**DOI:** 10.3389/fnsys.2021.805746

**Published:** 2022-01-25

**Authors:** Naoki Aizu, Ryoji Otaki, Kazuhiro Nishii, Takumi Kito, Runhong Yao, Kenya Uemura, Shin-ichi Izumi, Kouji Yamada

**Affiliations:** ^1^School of Health Sciences, Faculty of Rehabilitation, Fujita Health University, Toyoake, Japan; ^2^Department of Physical Medicine and Rehabilitation, Tohoku University Graduate School of Medicine, Sendai, Japan; ^3^Graduate School of Health Sciences, Fujita Health University, Toyoake, Japan; ^4^Department of Physical Therapy, School of Health Sciences, Japan University of Health Sciences, Satte, Japan; ^5^Department of Rehabilitation, Hachinohe City Hospital, Hachinohe, Japan; ^6^Department of Physical Medicine and Rehabilitation, Tohoku University Graduate School of Biomedical Engineering, Sendai, Japan

**Keywords:** body-specific attention, hand, foot, healthy adults, motor control, sensory function

## Abstract

To execute the intended movement, the brain directs attention, called body-specific attention, to the body to obtain information useful for movement. Body-specific attention to the hands has been examined but not to the feet. We aimed to confirm the existence of body-specific attention to the hands and feet, and examine its relation to motor and sensory functions from a behavioral perspective. The study included two groups of 27 right-handed and right-footed healthy adults, respectively. Visual detection tasks were used to measure body-specific attention. We measured reaction times to visual stimuli on or off the self-body and calculated the index of body-specific attention score to subtract the reaction time on self-body from that off one. Participants were classified into low and high attention groups based on each left and right body-specific attention index. For motor functions, Experiment 1 comprised handgrip strength and ball-rotation tasks for the hands, and Experiment 2 comprised toe grip strength involved in postural control for the feet. For sensory functions, the tactile thresholds of the hands and feet were measured. The results showed that, in both hands, the reaction time to visual stimuli on the hand was significantly lesser than that offhand. In the foot, this facilitation effect was observed in the right foot but not the left, which showed the correlation between body-specific attention and the normalized toe gripping force, suggesting that body-specific attention affected postural control. In the hand, the number of rotations of the ball was higher in the high than in the low attention group, regardless of the elaboration exercise difficulty or the left or right hand. However, this relation was not observed in the handgripping task. Thus, body-specific attention to the hand is an important component of elaborate movements. The tactile threshold was higher in the high than in the low attention group, regardless of the side in hand and foot. The results suggested that more body-specific attention is directed to the limbs with lower tactile abilities, supporting the sensory information reaching the brain. Therefore, we suggested that body-specific attention regulates the sensory information to help motor control.

## Introduction

To execute the intended movement, the brain generates a motor program based on various sensory information from the body. At that time, the brain directs attention to one’s own body and regulates the amount of information from the effectors to help with motor control. Recent research has shown that visual detection tasks can objectively measure attentional function. Specifically, responses to the visual stimuli on the body are faster than responses to the visual stimuli farther away from the body (Whiteley et al., 2004, 2008; Kao and Goodale, 2009) in the personal space, indicating that attention is potentially directed to the body (Aizu et al., 2018). This facilitation of response is observed not only in the personal space but also in the peripersonal space, near the body. Considering the peripersonal space, previous reports have demonstrated that healthy adults can detect visual targets more quickly when the targets are presented near the hand than when they are presented farther away (Reed et al., 2006, 2010; Dufour and Touzalin, 2008). This body facilitation effect, which enables faster detection of a target either on or nearer to the body, also occurs in situations involving proprioceptive information without hand visual information (Reed et al., 2006) and passive movement of the hand (Whiteley et al., 2008). This facilitation of response is based on the distance from the body (Hari and Jousmäki, 1996), suggesting that the response is faster on the body than near the body. This dominance of visual stimulus detection on the self-body was interpreted to be a result of latent attention towards the self-body, which is called body-specific attention (Aizu et al., 2018). Thus, it is important to understand the characteristics of this body-specific attention because sensory information from the extremities is useful for motor control.

Body-specific attention adapts to the activity status of the body. In patients with hemiparesis after a stroke, a time-dependent decline in body-specific attention to the paretic hand indicated that such decline was caused by patients learning to avoid using the paretic limb by considering the paretic limb useless for daily tasks rather than such decline being caused by brain damage (Aizu et al., 2018). Another study showed that detection improves for targets presented with the rake as a tool, after training with the rake in hand (Kao and Goodale, 2009; Reed et al., 2010). This finding provides strong support for the idea that this effect can be modulated by training, such that new objects, such as tools, can be incorporated into the body representation in the brain; that is, the brain recognizes tools as a part of the body. This affects the processing of visual stimuli (Kao and Goodale, 2009). Therefore, the facilitation either on or near the body is well documented in the context of body representations in the brain. Since peripersonal space in the foot reportedly facilitates the response (Schicke et al., 2009; Stettler and Thomas, 2017; Stone et al., 2018), it can be inferred that the personal space also facilitates the response in the foot, thereby indicating body-specific attention to the foot. However, Body-specific attention to the hands has been examined but not to the feet.

Owing to the different roles limbs play in human behavior (for example, hands are used for manipulating objects and feet are a means of postural control and transportation, and functional role of dominant and non-dominant in hand and foot), body-specific attention towards the hands and feet may be different. Considering the functional roles of the left and right feet, evidence indicates that the dominant foot produces a more propulsive force while the non-dominant foot provides preferential support during gait (Sadeghi et al., 1997). In addition, it is behaviorally unclear which functions are related to the body-specific attention to the hands and which to the feet. In the motor function of the hand, since body-specific attention plays an important role in motor control, we measured the ball rotation task as an elaborate movement requiring more control, and in contrast, we measured the simple handgrip task as maximal force exertion. In the motor function of the foot, since toe grasping force as foot motor function is an important factor for postural control (Kobayashi et al., 1999; Menz et al., 2005; Hashimoto and Sakuraba, 2014), we measured the grasping force; moreover, we measured the standing long jump task. In sensory function, many studies discussed that the tactile sensation is closely related to motor control. Previous reports have demonstrated that the precision grip of the hand and postural responses of the lower limb are impaired when sensory input from the hand (Johansson and Westling, 1984, 1987) or foot (Magnusson et al., 1990; Perry et al., 2000) is temporarily blocked in healthy participants. Recent reports in amputees have also demonstrated that generating tactile feedback through a prosthetic hand and foot can improve control with the prosthesis (Hebert et al., 2014; Valle et al., 2018; Petrini et al., 2019). Therefore, these findings indicated that sensory-motor integration plays an important role in motor control. In sensory function, we measured the tactile threshold of the hand and foot. We hypothesized that if body-specific attention exists, body-specific attention would be related to ball rotation tasks as elaborate movements in the hands, to toe grasping task in the feet in motor function, and body-specific attention would be related to the tactile threshold in sensory function. The purpose of this study is to confirm the existence of body-specific attention to the hands and feet and to examine how body-specific attention to the hands and feet is related to motor and sensory functions from a behavioral perspective.

## Methods

We performed a cross-sectional study using prospectively collected data from the Fujita Health University, Japan. Fifty-four healthy young adults participated in the experiment. Two groups of 27 individuals participated independently in two different experiments: Experiment 1 (mean age ± SD, 20.9 ± 0.5 years; 15 male, all with a right dominant hand) and Experiment 2 (mean age ± SD, 20.8 ± 0.7 years; 17 male, all with a right dominant foot; Oldfield, 1971; Chapman et al., 1987). Body-specific attention to the hands was measured in Experiment 1 and body-specific attention to the feet was measured in Experiment 2 by using a visual detection task. All the participants performed our visual detection task for the first time. We also assessed motor function using hand grip strength and a ball-rotation task in Experiment 1, and using toe grip strength and standing long jump tasks in Experiment 2. In addition, we assessed sensory function as the tactile threshold of the back of the hand in Experiment 1 and the sole of the foot in Experiment 2.

### Standard Protocol Approvals, Registrations, and Participants’ Consent

The Fujita Health University Ethics Committee approved this research (HM19-382), which was conducted in compliance with the ethical standards of the Declaration of Helsinki. Prior to our experiment, all participants agreed to participate in our experiment and provided written informed consent.

### Experimental Procedure

**Figure 1:**
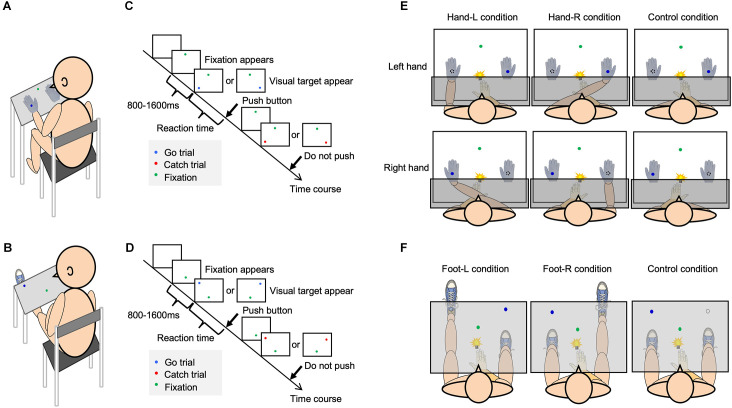
Experimental setup and the visual detection task. Each figure shows the experimental setup [**(A)** hand; **(B)** foot], procedure of visual detection task [**(C)** hand; **(D)** foot], and each condition [**(E)** hand; **(F)** foot].

In order to quantitatively measure body-specific attention to the hands and feet, a visual detection experiment was used for detecting light emitting diode (LED) lighting on either the surface of the self-body parts or surfaces far from the body parts (Figure 1). This experiment was designed to measure body-specific attention to detect a visual target near the body and has previously been tested in healthy adults (Whiteley et al., 2004, 2008; Reed et al., 2006, 2010; Kao and Goodale, 2009; Aizu et al., 2018). This task can measure body-specific attention as a body facilitation effect, which enables faster detection of a target on the body compared to a target far from the body.

The participants sat in a chair in front of a rack in a quiet room (Figures 1A,B). They were required to respond as quickly as possible to the glow of a blue LED that appeared on either the self-body parts or opposite to them. After the green LED fixation appeared, the target LED randomly appeared for 800–1,600 ms (Figures 1C,D). The participants were required to push the button only when a blue LED appeared, and gazed at the green LED as the fixation point. A blue LED representing a “go” visual target appeared in 80% of the trials. In the rest of the trials, a red LED representing a “no-go” target, that required no response, appeared. Reaction time (RT) was considered to be the time between the onset of glowing of the LED and the reaction of the participants (pushing the button). The response button was located at the same distance from each target LED. To equalize the visual information in the visual detection task, a white cloth or board covered the patients’ arms or legs (Figures 1E,F). In one condition, the participant performed 80 trials. Before the experiment, the participants performed 60 trials as a training session for the visual detection task. In order to eliminate the effects of delayed reactions owing to inattention during the task and accelerated reactions owing to anticipation of the appearance of the visual target, the obtained reaction time data that exceeded two standard deviations (SD) above and below the mean reaction time were excluded from the results as outliers.

### Conditions in Experiment 1

Body-specific attention to the left and right hands was measured according to previous methods (Aizu et al., 2018). To minimize differences in appearance between the self-hand and a dummy hand, both hands were covered with white cotton gloves (Figure 1E).

In the Hand-L condition, participants placed their hands in the left space; in contrast, a dummy hand was placed in the right space. In the Hand-R condition, the participants placed their hands in the right space; in contrast, a dummy hand was placed in the left space. In the control condition, the self-hand was placed on the abdomen, and two dummy hands were placed on the rack. The participants pushed a button with the index finger as soon as the target LED appeared. The participants were separately exposed to the tests in a counterbalanced manner.

The target LED was located 18 cm from the midsagittal plane. The distance between the green LED and target LED was 28 cm, and the distance between the target LED and the participants was 28 cm. The dummy hand was placed 36 cm away from the hand on the rack.

### Conditions in Experiment 2

To measure body-specific attention to the left and right foot, the conditions were defined as the Foot-L, Foot R, and Control conditions (Figure 1F). In the Foot-L condition, the participant placed their left foot on the foot stand (knee extension position), and the right foot was placed on the ground. The left foot was positioned such that the target LED was located at the center of the left ankle. In the Foot-R condition, the participant placed their right foot on the foot stand (knee extension position), with the right foot positioned where the target LED on the right side was presented. The left foot was placed on the ground. In the control condition, the left and right feet were both on the ground, and participants responded to the target LED to confirm the attentional bias between the left and right spaces. The participants pushed a button with their right index finger as soon as the target LED appeared. The participants were separately exposed to the tests in a counterbalanced manner.

At the beginning of each trial, the target LED was located 21 cm from the midsagittal plane, the distance between the green LED and the target LED was 32 cm, and the distance between the target LED and the participants was approximately 65 cm, depending on the length of their lower extremities.

### Motor Assessment

In Experiment 1 relating to the hands, we conducted a ball-rotation task and a handgrip task for motor function measurements. In the ball-rotation task, they rotated two balls using either their left or right hand to assess the elaborate movement. The two balls were rotated on the palmar surface for 20 s. The measurements were recorded using a video camera, and the number of rotations of the balls were counted. The balls were spherical, 4 cm in diameter, and weighed 75 g. To confirm whether the ball rotation task was an elaborate movement for the participants, two tasks with different difficulty levels were performed: the easy task was clockwise rotation with the right hand and counterclockwise rotation with the left hand, while the hard task required the right hand to rotate the ball counterclockwise and the left hand to rotate it clockwise. Participants performed a handgripping task to assess the maximal force exertion in the left and right hands.

In Experiment 2, we conducted a toe grip strength task and a standing long jump task. In relation to the feet, the participants performed the toe gripping task (Toe grip dynamometer, Takei Scientific Instruments) to assess the maximum force exerted in the left and right feet. In the standing long jump task, they jumped as far as they could from a standing position and the distance was measured. All motor assessments were performed thrice, and the average of the three assessments was calculated. In addition, for each task, the trial order of the left and right, and the difficulty level were counterbalanced for each participant.

### Sensory Assessment

We measured the tactile threshold using Semmes–Weinstein monofilaments (Sakai Medical) of different thicknesses. We measured the tactile thresholds of the dorsum of the hand (second metacarpal metaphyseal part) in Experiment 1 and the sole of the foot (ball of the big toe) in Experiment 2. The staircase method was used for the measurement, and the average value of seven change points was calculated as the tactile threshold. The left and right trial orders were counterbalanced and conducted for each participant.

### Statistical Analyses

The measured data were checked for normality using the Shapiro–Wilk test before the usage of statistical methods. To confirm body-specific attention to the hand in Experiment 1, we performed a three-way analysis of variance (ANOVA; hand, hand position, target LED position) with repeated measures and multiple comparisons, as a *post hoc* test with the Bonferroni correction. If there was an interaction effect between the two factors (hand position and visual target position), the response to visual stimuli on the hand was significantly faster than that to stimuli on the fake hand. To confirm body-specific attention to the foot in Experiment 2, we performed a two-way analysis of variance (ANOVA; foot position, target LED position) with repeated measures and multiple comparisons, as a *post hoc* test with the Bonferroni correction. If there was an interaction effect between the two factors (foot position and target LED position), the response to visual stimuli on the foot was significantly faster than that to stimuli off the foot. In the control condition, we used two-way ANOVA with repeated measures and paired t-tests to compare the response times of both sides in Experiments 1 and 2, respectively.

The index of body-specific attention was calculated by subtracting the RT for the target-on-self-body parts from that for the target-off-self-body parts. Furthermore, to examine what attention to the hand and foot affected participant-specific factors, we classified the participants into low and high attention groups based on the index of body-specific attention to each left and right hand and foot. In the comparison between the two groups, the results of the motor and sensory assessment were compared by using the three-way and two-way ANOVA.

To avoid the effect of body size on the maximal force exertion in the hand and foot, the measured values were divided by body weight and normalized. In addition, the score of the standing long jump was normalized according to the body height. Statistical significance was set at *p* < 0.05. Regarding the software for analysis, we used SPSS version 26.

## Results

**Figure 2:**
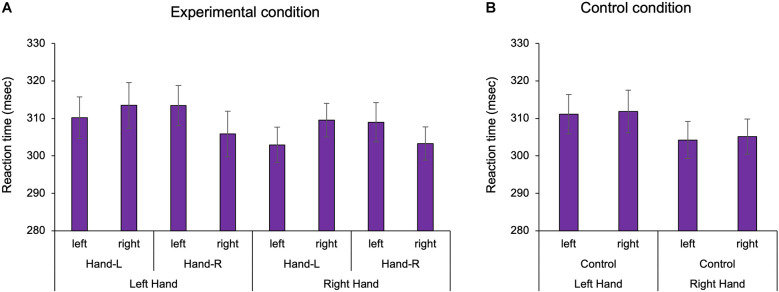
The reaction time of the hand in Experiment 1. In the experimental condition, the existence of the hand facilitated the reaction time in the left and right hands, regardless of hand position. In the control condition, there was no difference in the reaction time between the left and right sides. Hand-L: The participant placed their hand in the left space. Hand-R: The participant placed their hand in the right space. **(A)** Experimental condition; **(B)** Control condition, Mean ± Standard error.

**Figure 3:**
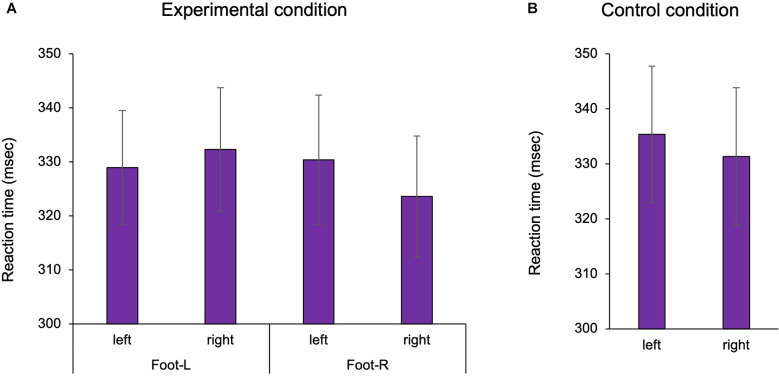
The reaction time of the foot in Experiment 2. In the experimental condition, the presence of the foot facilitated the reaction time. In the control condition, there was no difference in the reaction time between the left and right sides. Foot-L: The left foot was positioned under the left target LED. Foot-R: The right foot was positioned under the right target LED. **(A)** Experimental condition; **(B)** Control condition, Mean ± Standard error. LED, light emitting diode.

We confirmed the existence of body-specific attention to the hands and feet in Experiments 1 and 2. In the visual detection task, the three-way ANOVA with repeated measures showed a statistically significant interaction term in Experiment 1 (Two factors: hand position, visual target position *F*_(1,25)_ = 32.609, *p* < 0.001, $\eta_{p}^{2}$ = 0.538; Figure 2). In the multiple comparisons with the Bonferroni correction, the RT on the hand was shorter than that off the hand in the hand-L (*p* = 0.011) and hand-R conditions (*p* = 0.008). RT was facilitated when the hand position and the visual target position were spatially congruent than when incongruent, regardless of the hand. The ANOVA of healthy adults showed that there was no main effect for the factors (hand: *F*_1,25_ = 2.184, *p* = 0.152, $\eta_{p}^{2}$ = 0.050 hand position: *F*_1,25_ = 0.771, *p* = 0.388, $\eta_{p}^{2}$ = 0.010, visual target position *F*_1,25_ = 0.001, *p* = 0.983, $\eta_{p}^{2}$ = 0.008) and no interaction effect (hand × hand position × visual target position: *F*_1,25_ = 0.005, *p* = 0.942, $\eta_{p}^{2}$ = 0.007). In the control condition, the two-way ANOVA with repeated measures showed that there was no main effect for the factors (hand: *F*_1,26_ = 2.326, *p* = 0.139, $\eta_{p}^{2}$ = 0.082, visual target position: *F*_1,26_ = 0.150, *p* = 0.701, $\eta_{p}^{2}$ = 0.006) and no interaction (*F*_1,26_ = 0.003, *p* = 0.957, $\eta_{p}^{2}$ = 0.001). In other words, there was no difference in the RT for the left and right sides in Experiment 1. For Experiment 2, a two-way ANOVA with repeated measures showed a statistically significant interaction term (two factors: foot position, visual target position, *F*_1,26_ = 4.936, *p* = 0.035, $\eta_{p}^{2}$ = 0.160; Figure 3). In the multiple comparisons with the Bonferroni correction, the RT on the foot was shorter than that off the foot in the foot-R conditions (*p* = 0.026), while in the foot L conditions, there was no significance (*p* = 0.306). In addition, the ANOVA for healthy adults showed that there was no main effect for the factors (foot position *F*_1,26_ = 1.544, *p* = 0.225, $\eta_{p}^{2}$ = 0.056, visual target position *F*_1,26_ = 0.745, *p* = 0.398, $\eta_{p}^{2}$ = 0.028). In the control condition, there was no difference in the RT between the left and right sides in Experiment 2 (paired t-test, df = 26 *t* = 1.674, *p* = 0.106). In the right foot, RT was facilitated when the foot position and the visual target position was spatially congruent than when incongruent. The average incorrect responses in Experiment 1 were 0.44 times in the “no-go” target (16 trials) and 0.40 in Experiment 2, showing no difference in the incorrect response times between Experiments 1 and 2.

**Figure 4:**
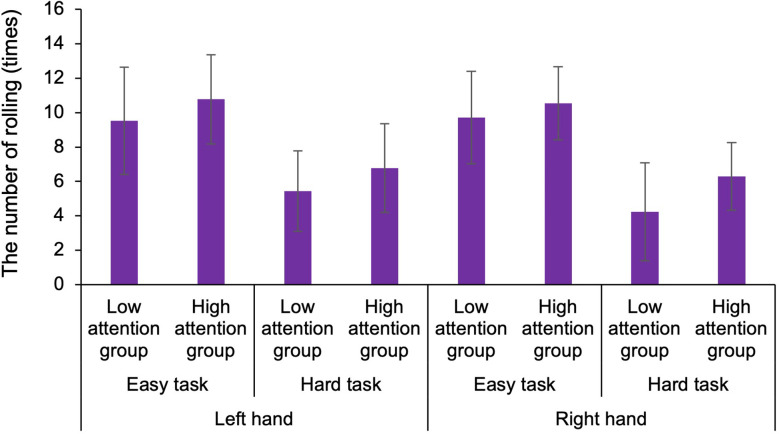
Comparison of the number of rotations of the ball for different difficulty tasks in the low and high attention groups. The number of rotations of the ball was higher in the high attention group than that in the low attention group, regardless of the difficulty of the elaboration exercise or the left or right hand. The level of difficulty was distinguished between the hard and easy tasks.

**Figure 5:**
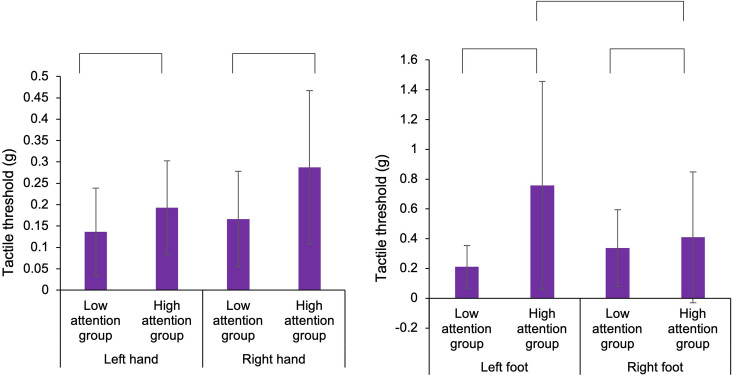
Comparison of the tactile threshold of the hands and feet in the low and high attention groups. In the hands and feet, the tactile threshold in the high attention group was higher than that in the low attention group. In the foot, in the high attention group, the tactile threshold in the left foot was higher than that in the right foot.

To examine the functions of body-specific attention in healthy adults, we compared the results of the motor and sensory assessments by classifying participants into low and high attention groups based on the index of body-specific attention. For the hand, in ball rotation task, three-way ANOVA demonstrated a statistically significant main effect for the factors (task: *F*_1,50_ = 81.139, *p* < 0.001, $\eta_{p}^{2}$ = 0.448, groups: *F*_1,50_ = 7.593, *p* = 0.007, $\eta_{p}^{2}$ = 0.071; Figure 4). Moreover, there was no main effect for the factors (hand: *F*_1,50_ = 0.764, *p* = 0.384, $\eta_{p}^{2}$ = 0.008) and no interaction effect (hand × task × groups: *F*_1,50_ = 0.337, *p* = 0.563, $\eta_{p}^{2}$ = 0.003). Thus, the number of rotations of the ball was higher in the high attention group (left hand in easy task: Mean = 10.7, SD = 2.6, *N* = 13, left hand in hard task: Mean = 6.8, SD = 2.9, *N* = 13, right hand in easy task: Mean = 10.5, SD = 2.1, *N* = 13, right hand in hard task: Mean = 6.3, SD = 1.9, *N* = 13) than that in the low attention group (left hand in easy task: Mean = 9.5, SD = 3.1, *N* = 14, left hand in hard task: Mean = 5.4, SD = 2.3, *N* = 14, right hand in easy task: Mean = 9.7, SD = 2.7, *N* = 14, right hand in hard task: Mean = 6.2, SD = 2.8, *N* = 14), regardless of the difficulty of the elaboration exercise or the left or right hand. In addition, our results confirmed that the level of difficulty was distinguished between the hard and the easy task. In the normalized gripping force of gripping task, two-way ANOVA demonstrated that there was no main effect (hand: *F*_1,50_ = 0.835, *p* = 0.365, $\eta_{p}^{2}$ = 0.016, groups: *F*_1,50_ = 3.331, *p* = 0.075, $\eta_{p}^{2}$ = 0.062) and no interaction (hand × groups: *F*_1,50_ = 0.285, *p* = 0.596, $\eta_{p}^{2}$ = 0.006). In the tactile sensation of the hand, the tactile threshold in the high attention group (left hand: Mean = 0.193, SD = 0.109, *N* = 13, right hand: Mean = 0.288, SD = 0.179, *N* = 13) was higher than that in low attention group (left hand: Mean = 0.136, SD = 0.102, *N* = 14, right hand: Mean = 0.166, SD = 0.111, *N* = 14), regardless of the left or right hand (Figure 5). Two-way ANOVA showed a statistically significant main effect for the factor (groups: *F*_1,50_ = 6.466, *p* = 0.014, $\eta_{p}^{2}$ = 0.115). Moreover, there was no main effect (hand: *F*_1,50_ = 3.172, *p* = 0.081, $\eta_{p}^{2}$ = 0.060) and interaction (hand × groups: *F*_1,50_ = 0.853, *p* = 0.360, $\eta_{p}^{2}$ = 0.017).

**Figure 6:**
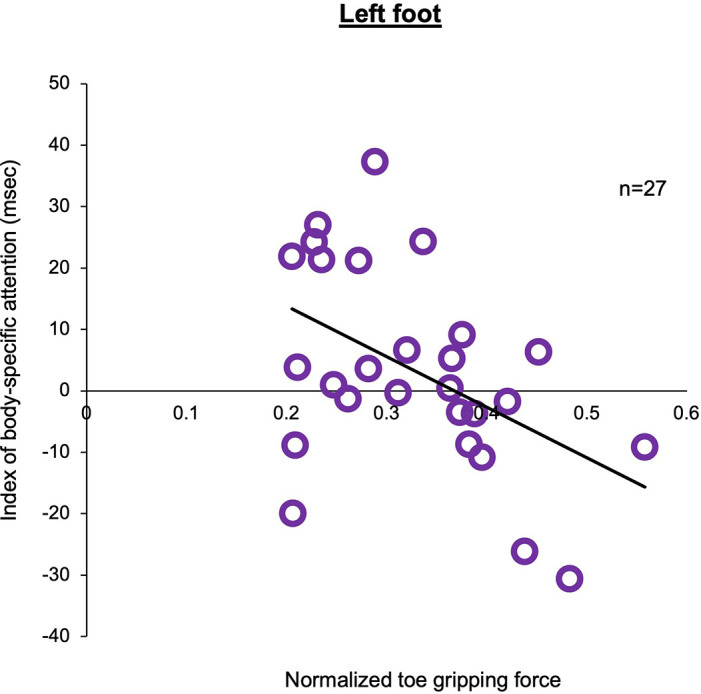
Correlation between the index of body-specific attention to the foot and normalized toe gripping force in the left foot.The higher the value of the normalized toe gripping force, the stronger the toe gripping force against body weight. There was a negative correlation between the index of body-specific attention to the foot and normalized toe gripping force (Pearson, *n* = 27, *r* = −0.476, *p* = 0.012).

In experiment 2, in tactile sensation of the foot, the tactile threshold in the high attention group (left foot: Mean = 0.758, SD = 0.698, *N* = 13, right foot: Mean = 0.409, SD = 0.439, *N* = 13) was higher than that in the low attention group (left foot: Mean = 0.211, SD = 0.142, *N* = 14, right hand: Mean = 0.337, SD = 0.258, *N* = 14), regardless of the left or right foot (Figure 5). Two-way ANOVA showed a statistically significant main effect for the factor (groups: *F*_1,50_ = 6.968, *p* = 0.011, $\eta_{p}^{2}$ = 0.122) and interaction (foot × groups: *F*_1,50_ = 4.103, *p* = 0.048, $\eta_{p}^{2}$ = 0.076). Moreover, there was no main effect (foot: *F*_1,50_ = 0.900, *p* = 0.347, $\eta_{p}^{2}$ = 0.018). In multiple comparisons with the Bonferroni correction, the tactile threshold of the high attention group in left foot was higher than that of the low attention group in the left foot (*p* = 0.002) and that of the high attention group in the right foot (*p* = 0.044). In normalized distance of the long jump task, two-way ANOVA showed that there was no main effect (foot: *F*_1,50_ = 0.001, *p* = 0.995, $\eta_{p}^{2}$ = 0.001, groups: *F*_1,50_ = 1.701, *p* = 0.198, $\eta_{p}^{2}$ = 0.033) and no interaction (hand × groups: *F*_1,50_ = 0.033, *p* = 0.856, $\eta_{p}^{2}$ = 0.001). In the normalized toe gripping force of the foot gripping task, two-way ANOVA showed that there was interaction (foot × groups: *F*_1,50_ = 4.609, *p* = 0.037, $\eta_{p}^{2}$ = 0.084), but no main effect (foot: *F*_1,50_ = 0.559, *p* = 0.458, $\eta_{p}^{2}$ = 0.011, groups: *F*_1,50_ = 0.125, *p* = 0.725, $\eta_{p}^{2}$ = 0.003). In the multiple comparisons with the Bonferroni correction, the normalized toe gripping force of the high attention group in the left foot tend to be lower than that of the low attention group in the left foot (*p* = 0.083) and that of the high attention group in the right foot (*p* = 0.050). In the left foot, no facilitation of the reaction time was observed when the foot position and the visual target position were spatially congruent; however, there was a relationship between body-specific attention and the normalized toe gripping force. Therefore, a correlation analysis between body-specific attention and normalized toe gripping force was conducted. In the correlation analysis, there was a correlation between body-specific attention of the left foot and the normalized toe gripping force of the left foot (Pearson, *r* = −0.476, *p* = 0.012; Figure 6).

## Discussion

In healthy adults, we confirmed the existence of body-specific attention to the hands and feet. In Experiment 1, the number of rotations of the ball was higher in the high attention group than that in the low attention group, regardless of the difficulty of the elaboration exercise or the left or right hand. However, this relation was not observed in the gripping task. These findings indicated that body-specific attention of the hand is an important factor in the motor control as elaborate movement. In the tactile sensation of the hand and foot, the tactile threshold in the high attention group was higher than that in the low attention group, regardless of the left or right. The results suggested that more body-specific attention is directed to the limbs with lower tactile abilities, supporting the sensory information reaching the brain. In Experiment 2, in the visual detection task, the facilitation effect of the reaction time was observed in the right foot but not in the left foot. In the left non-dominant foot, which plays an important role in postural control, there was a correlation between body-specific attention of the left foot and the normalized toe gripping force of the left foot, indicating that body-specific attention was affected by the toe grip strength in the left foot. In the right dominant foot, on the other hand, there was no relationship between the body-specific attention and other motor functions. Thus, body-specific attention to the feet demonstrated differences between the left and right sides, suggesting that body-specific attention varied based on the functional role of the left and right foot. Considering that body-specific attention adapts to the state of the body (Aizu et al., 2018), we indicated that body-specific attention to the hands and feet plays an important role in motor control. Therefore, we suggested that body-specific attention regulates the sensory information to help motor control.

Our results indicate that body-specific attention supports elaborate movements. Previous reports have shown that spatial selective attention to the body facilitates somatosensory information processing (García-Larrea et al., 1991; Schubert et al., 2008). The response in the primary somatosensory cortex is generally gated during simple movement of the corresponding body part (Nakata et al., 2003; Wasaka et al., 2003), but elaborate movements such as ball rotation showed an enhancement of sensorimotor integration in the somatosensory cortex compared to other simple tasks such as grasping (Wasaka et al., 2017). In our results, for the left and right hand, the number of ball rotations was high in the high attention group. This result was also observed in the easy and difficult ball-rotation tasks. In addition, there was no relationship between the scores for body-specific attention and handgrip strength. Interestingly, our data showed that tactile thresholds were higher in the high attention group. Therefore, for participants who could not discriminate between small amounts of tactile stimuli, adaptive changes were suggested to increase sensory input to the brain by directing attention to their own hand. Past reports showed that elaborate movements are impaired when sensory input from the hand is temporarily blocked in healthy adults (Johansson and Westling, 1984, 1987). These findings suggest that body-specific attention facilitates sensory information processing during elaborate movement and facilitates motor control.

In Experiment 2, the visual stimulus detection task showed the presence of body-specific attention in the right foot but not in the left foot. Similar to our results, in the peripersonal space of the foot, the facilitation effect was observed only in the right foot and not in the left foot (Stettler and Thomas, 2017). Importantly, in the left foot, our correlation results showed that weak toe grip strength resulted in a higher score of body-specific attention to the foot. A previous study reported that toe plantar flexor muscle strength was one of the predictors of balance ability in older adults (Menz et al., 2005). This study revealed that weak toe flexor muscle strength indicates poor balance ability. In addition, preliminary evidence suggests that “grasping” exercises to strengthen toe muscles result in improved standing balance in older adults (Kobayashi et al., 1999). Moreover, in young adults, training the foot flexor strength improves movement performance (Hashimoto and Sakuraba, 2014) and gait ability (Fukuda and Kobayashi, 2008). These findings indicate that toe grasping force is an important factor in postural control. In addition, the participants in this study were all right-footed according to the dominant foot test (Chapman et al., 1987), indicating that the right foot was the dominant foot. In the functional role of the left and right feet, there is evidence suggesting that the dominant foot generates more propulsion during walking, while the non-dominant foot preferentially provides support (Sadeghi et al., 1997). Martelli et al. (2013) suggested that the non-dominant foot may be better suited for maintaining stability in response to perturbations. These findings indicate that the left foot has a higher postural control ability to support and maintain the body than the right foot (dominant foot). This could explain our results, which demonstrated a strong relationship between toe grip strength and body-specific attention only in the left foot. Considering that body-specific attention adapts to the activity status of the body (Aizu et al., 2018), in the left foot, we suggested that body-specific attention would be adaptively altered by the toe grip strength. Therefore, there is an adaptive change that increases the body-specific attention when toe grip strength is weak, and this adaptive change presumably occurs to help postural control.

Our data demonstrated that the tactile threshold was higher in the high attention group in both feet. Importantly, previous reports have shown impaired postural responses when sensory input is blocked on the foot in healthy persons (Magnusson et al., 1990; Perry et al., 2000). Given that the facilitates sensory information processing by attention (García-Larrea et al., 1991; Schubert et al., 2008), we suggested that when the function of sensory discrimination of the feet is low, body-specific attention may increase the sensory input to the brain. Therefore, these findings suggested that body-specific attention facilitates sensory information processing during postural control in the foot. However, body-specific attention to the right foot did not relate toe gripping task and standing long jump task in this study. Further investigation of the relationship between body-specific attention to the foot and postural control is warranted.

We have several hypotheses regarding the neural basis of body-specific attention. First, recent studies related to body representation in the brain have focused on the frontoparietal network (Naito et al., 2007; Takeuchi et al., 2016), and we hypothesize that body-specific attention reflecting body representation in the brain is related to this network. This network includes brain regions related to the body in the brain, including body consciousness, which includes a sense of ownership and a sense of agency (Ehrsson et al., 2004; Naito et al., 2007; Gentile et al., 2013; Ohata et al., 2020). This frontoparietal network represents the self-body in the brain and contributes to the realization of efficient motor control and body cognition in humans. Furthermore, this frontoparietal network has also been previously known as the attentional network, which plays an important role in understanding the positional relationship between the body and the environment (Buschman and Miller, 2007; Cona and Scarpazza, 2019). These findings suggest that the neural basis of body-specific attention is most likely a frontoparietal network for attention and body representations in the brain. Next, because body-specific attention is measured based on the effect of nearby-hands (Reed et al., 2010; Tseng et al., 2012), it is likely that the posterior parietal cortex (PPC), which is the brain’s region of peripersonal spatial representation, is the most important part of the frontoparietal network (Moseley et al., 2012). The PPC is a region of multisensory integration, which includes vision and somatosensory perception, and is classically known as a region involved in body schema and body image. Although most studies related to self-body have been conducted on the upper limbs, the results of studies on the lower limbs suggest a similar neural basis. Contrarily, studies on healthy subjects and amputees have demonstrated that somatotopy occurs in the sensorimotor cortex before input to the parietal association cortex (Lotze et al., 2001; Simões et al., 2012). In the hierarchy of perceptual representation, the body part is reproduced clearly, and at a higher level of propositional representation, the body part is modified *via* top-down awareness of the environment and contextual thoughts, and the integrated self-body representation is used to carry out daily activities (Cona and Scarpazza, 2019). We believe that the neural basis of body-specific attention involves the frontoparietal network, with the parietal lobe being a particularly important region.

There are limitations to this study as well. First, the difference in motor and sensory functions in healthy adults is very small compared to that in patients with motor or sensory dysfunction and top athletes or skilled performers in music. By measuring body-specific attention in these participants, future studies will be able to clarify the characteristics of body-specific attention. In addition, interventions on body-specific attention may be able to improve motor performance. Second, our results could not clearly demonstrate the relationship between body-specific attention and motor control of the foot. It is necessary to investigate body-specific attention and motor function, especially postural control, taking into account functional differences between the left and right in the foot.

In summary, this study showed the presence of body-specific attention to the feet and hands. In addition, body-specific attention was shown to be closely related to motor and sensory functions. Furthermore, it was shown that body-specific attention is adaptively altered to obtain the sensory information necessary for motor control. Given these adaptive changes in body-specific attention, further research is needed to clarify the role of body-specific attention in improving motor function.

## Data Availability Statement

The datasets presented in this study can be found in online repositories. The names of the repository/repositories and accession number(s) can be found below: https://doi.org/10.5061/dryad.9ghx3ffjc.

## Ethics Statement

The studies involving human participants were reviewed and approved by The Fujita Health University ethics committee in Fujita Health University (HM19-382). The patients/participants provided their written informed consent to participate in this study.

## Author Contributions

NA conceived the study concept and design and wrote the manuscript. NA, TK, RY, and KN collected data. NA, RO, and KU analyzed and interpreted the data. S-iI and KY supervised the study. All authors reviewed the manuscript. All authors contributed to the article and approved the submitted version.

## Conflict of Interest

The authors declare that the research was conducted in the absence of any commercial or financial relationships that could be construed as a potential conflict of interest.

## Publisher’s Note

All claims expressed in this article are solely those of the authors and do not necessarily represent those of their affiliated organizations, or those of the publisher, the editors and the reviewers. Any product that may be evaluated in this article, or claim that may be made by its manufacturer, is not guaranteed or endorsed by the publisher.
